# Individual prediction of psychotherapy outcome in posttraumatic stress disorder using neuroimaging data

**DOI:** 10.1038/s41398-019-0663-7

**Published:** 2019-12-02

**Authors:** Paul Zhutovsky, Rajat M. Thomas, Miranda Olff, Sanne J. H. van Rooij, Mitzy Kennis, Guido A. van Wingen, Elbert Geuze

**Affiliations:** 1 Department of Psychiatry, Amsterdam UMC, Location AMC, University of Amsterdam, Amsterdam Neuroscience, Amsterdam, The Netherlands; 2 Amsterdam Brain and Cognition, University of Amsterdam, Amsterdam, The Netherlands; 3 ARQ National Psychotrauma Centre, Diemen, The Netherlands; 4 Department of Psychiatry and Behavioral Sciences, Emory University School of Medicine, Atlanta, GA USA; 5 Clinical Psychology Department, Utrecht University, Utrecht, The Netherlands; 6 Utrecht University Medical Center, Rudolf Magnus Institute of Neuroscience, Utrecht, The Netherlands; 7 Brain Research and Innovation Centre, Ministry of Defence, Utrecht, The Netherlands

**Keywords:** Neuroscience, Psychology

## Abstract

Trauma-focused psychotherapy is the first-line treatment for posttraumatic stress disorder (PTSD) but 30–50% of patients do not benefit sufficiently. We investigated whether structural and resting-state functional magnetic resonance imaging (MRI/rs-fMRI) data could distinguish between treatment responders and non-responders on the group and individual level. Forty-four male veterans with PTSD underwent baseline scanning followed by trauma-focused psychotherapy. Voxel-wise gray matter volumes were extracted from the structural MRI data and resting-state networks (RSNs) were calculated from rs-fMRI data using independent component analysis. Data were used to detect differences between responders and non-responders on the group level using permutation testing, and the single-subject level using Gaussian process classification with cross-validation. A RSN centered on the bilateral superior frontal gyrus differed between responders and non-responder groups (*P*_FWE_ < 0.05) while a RSN centered on the pre-supplementary motor area distinguished between responders and non-responders on an individual-level with 81.4% accuracy (*P* < 0.001, 84.8% sensitivity, 78% specificity and AUC of 0.93). No significant single-subject classification or group differences were observed for gray matter volume. This proof-of-concept study demonstrates the feasibility of using rs-fMRI to develop neuroimaging biomarkers for treatment response, which could enable personalized treatment of patients with PTSD.

## Introduction

Posttraumatic stress disorder (PTSD) is a psychiatric disorder that can develop after experiencing a traumatic event. It is characterized by states of re-experiencing of the traumatic event, avoidance of trauma-reminders, emotional numbing, and hyperarousal^1^. PTSD lifetime prevalence rates in the general population are estimated to be below 10% (varying between 1.3 to 8.8% depending on the country)^2,3^ but can vary heavily in veterans (between 1.4 to 31%)^4,5^. Treatment of PTSD typically involves trauma-focused psychotherapy with or without the administration of medication such as selective serotonin reuptake inhibitors (SSRIs). Trauma-focused therapies, such as trauma-focused cognitive behavior therapy (TF-CBT) or eye movement desensitization and reprocessing (EMDR) have been suggested as first-line treatments for treating PTSD^6,7^. However, 30–50% of patients do not benefit sufficiently^8^. To improve treatment response rates it is important to better understand differences between responders and non-responders, and identify reliable predictors for treatment outcome.

PTSD is characterized as a brain disorder showing alterations in activity and connectivity of cortical and sub-cortical brain regions. The neurocircuitry model of PTSD suggests that PTSD pathology is characterized by hyperactivity and increased connectivity of the amygdala, the anterior insula and the anterior cingulate cortex, decreased activity of the ventromedial prefrontal cortex (vmPFC) and hypoconnectivity between vmPFC, hippocampus and amygdala^9–12^. Functional connectivity of these regions can be recorded using neuroimaging techniques such as resting-state functional magnetic resonance imaging (rs-fMRI). Therefore, it is important to investigate if those alterations in rs-fMRI connectivity could be used to predict treatment-outcome and reveal biomarkers to increase the treatment-response rate. Indeed, pre-treatment group differences in fMRI activity and connectivity were observed between responders and non-responders in PTSD in several studies^13–17^. However, these group-level univariate analyses focus on average differences between responders and non-responders. This does not allow inference at the individual patient level, which can be achieved using multivariate supervised machine learning analyses^18,19^. Most importantly, performance is evaluated on new data to estimate the generalizability of the trained models, and thereby enabling the prediction of treatment outcome for new patients. Machine-learning analyses have been performed in the context of PTSD using different modalities of MRI data to distinguish between patients and controls^20–23^. However, only two studies to date have used machine learning analyses to predict future outcome at an individual level. One study aimed to predict clinical status two years after treatment with 12 weeks of paroxetine in a sample of 20 civilian PTSD patients^24^. This study used pre- and post-treatment rs-fMRI derived measures, namely amplitude of low-frequency fluctuations and whole-brain degree centrality maps, and the results showed that pre- but not post-treatment measures were able to predict remission status after two years with an accuracy of 72.5%. But as all but one patient had been in remission shortly after treatment, these results reflect relapse rather than treatment outcome. In addition, one recent study used a combination of resting-state connectivity within the ventral attention network and delayed recall performance in a verbal memory task to predict the response to prolonged exposure therapy in ~19 civilian patients with PTSD^25^. Although the proportion of treatment non-responders was low, the classifier still managed to distinguish the groups with ≥80% sensitivity and specificity.

To determine whether neuroimaging data could also predict treatment outcome in a larger sample of combat veterans with PTSD, we analyzed pre-treatment structural MRI and rs-fMRI data of 44 patients who received treatment-as-usual. This consisted of trauma-focused psychotherapy such as TF-CBT and EMDR, and clinical outcome was determined 6–8 month following the baseline fMRI scan. We previously reported pre-treatment group differences in structural^26^, white-matter^27^ and task-based (f)MRI^13,28^ between responders and non-responders, as well as rs-fMRI differences between patients and controls^29^. In another study, we explored time-by-group interactions between remitted and persistent PTSD patients based on network measures derived from rs-fMRI^30^. In the current study, we focus on pre-treatment MRI measures exclusively within a machine-learning paradigm. We extracted functional connectivity (FC) within resting-state networks (RSNs) using independent component analysis (ICA). ICA was chosen as the method for analyzing rs-fMRI because it provides a multivariate and data-driven way to identify multiple RSN’s present in the data. It does not require the definition of seed regions and is more robust to noise^31^. To ensure independence between RSN identification and estimation of RSN expression for each individual patient, the ICA was performed on rs-fMRI data of sex and age matched combat controls (*n* = 28). In addition, we derived maps of regional gray matter volume using voxel-based morphometry (VBM). Abnormalities in structural MRI data have been repeatedly linked to treatment-response in PTSD^26,32–35^ and we therefore used it as a standardized and easy to acquire imaging baseline. For both the rs-fMRI and VBM data, we performed univariate inference on the group level as well as multivariate prediction on the single-subject level using Gaussian process classification (GPC) with 10 × 10 cross-validation. The current study is exploratory and investigated the general feasibility of structural and rs-fMRI data to predict treatment-outcome in PTSD on the single-subject level. Because of its exploratory nature, there were no a priori hypotheses formulated.

## Materials and methods

### Participants

In total 57 veterans with PTSD and 29 combat controls (CC) were included in the study. Patients were recruited from one of four outpatient clinics of the Military Mental Healthcare Organization in Utrecht, The Netherlands. PTSD diagnosis was established by a licensed psychologist or psychiatrist. The Clinician Administered PTSD scale (CAPS)^36^ for DSM-IV^1^ was administered by trained research staff to quantify the total symptom severity and had to be ≥45. Combat controls had to have no current psychiatric disorders and a total CAPS score < 15. Further inclusion criteria for all subjects were deployment to a war zone and 18–60 years of age. Comorbid disorders were examined using the structured clinical interview for DSM-IV (SCID-I)^37^. Subjects with a history of neurological disorders, current substance dependence and contraindications for MRI scanning were excluded. From the initial 57 PTSD patients, seven were lost to follow-up, three were excluded based on excessive motion during scanning (see Supplementary information), one due to an artifact in the MRI scan, and one due to refusal of scanning. One additional participant was excluded as she was the only female in the sample. This lead to the final sample of 44 PTSD patients. From the CC only one subject had to be excluded based on excessive motion (*n* = 28).

After a period of six to eight months in which patients underwent treatment-as-usual consisting of trauma-focused therapy (e.g., TF-CBT, EMDR) a second CAPS assessment was performed. Treatment response was defined as a ≥30% decrease of total CAPS score at follow-up with respect to the baseline assessment^38,39^. According to this criterion 24 PTSD veterans were defined as responder and 20 as non-responder. All participants gave written informed consent. The study was approved by the University Medical Center Utrecht ethics committee, in accordance with the declaration of Helsinki^40^.

### Clinical data analysis

To estimate whether the CC, responders and non-responders differed across any demographic or clinical variables at baseline or follow-up ANOVA, Kruskal–Wallis, *χ*^2^, or *t*-tests were applied as appropriate. All tests were performed using the R software (version 3.5.1).

### Data acquisition

All scans were obtained on a 3T MRI scanner (Philips Medical System, Best, the Netherlands). The T1-weighted high resolution MRI scan was acquired before the rs-fMRI scan with the following parameters: repetition time (TR) = 10 ms, echo time (TE) = 4.6 ms, flip angle = 8°, 200 sagittal slices, field of view (FOV) = 240 × 240 × 160, matrix size = 304 × 299 and voxel size = 0.8 × 0.8 × 0.8 mm. The rs-fMRI scan consisted of 320 T2*-weighted echo planar interleaved slices with TR = 1600 ms, TE = 23 ms, flip angle = 72.5°, FOV = 256 × 208 × 120, 30 transverse slices, matrix size = 64 × 51, total scan time 8 min and 44.8 s, 0.4 mm gap, acquired voxel size = 4 × 4 × 3.60 mm). Participants were asked to focus on a fixation cross, while letting their mind wander and relax.

### MRI data preprocessing

To estimate whether structural images carry information to distinguish between responders or non-responders a VBM analysis was performed. Gray matter (GM) voxel-wise volume maps were computed using the SPM12 toolbox (v7219, https://www.fil.ion.ucl.ac.uk/spm/software/spm12/).

Resting-state fMRI images were preprocessed using the advanced normalization tools (ANTs, 2.1.0, http://stnava.github.io/ANTs/) and FMRIB Software Library (FSL, 5.0.10, https://fsl.fmrib.ox.ac.uk/fsl/fslwiki/. To control for the influence of motion on the rs-fMRI data ICA-AROMA was applied^41^. Details on the preprocessing pipelines can be found in the Supplementary information.

### Resting-state network identification

Preprocessed rs-fMRI data were analyzed to determine group-level resting-state networks (RSNs). Group components with a fixed number of 70 components were estimated using a meta-ICA approach utilizing FSL’s MELODIC software^42^ applied to the rs-fMRI data of the CC. The identification of RSNs only on the data of the CC was done to reduce the potential of overfitting during the machine learning analysis since the training and test data sets would have to be taken together when defining the RSNs if data of the PTSD patients would be included. However, a potential drawback is that the identified RSNs might not optimally represent the rs-fMRI of the PTSD patients. The number of components was fixed to 70 because it was shown to provide good insight into clinical differences of patient groups^43^. However, it should be noted that such a higher-order ICA can split up canonical networks into multiple sub-networks which might reduce the information present in each sub-network. The meta-ICA approach allows for the identification of reproducible and reliable group components^44^. After meta-ICA, 48 RSN’s were identified using a semi-automatic approach. Thereafter, FSL’s dual regression approach was used to estimate single-subject spatial representations of the corresponding group networks for all patients. Details on the implementation and rationale of the procedure can be found in the Supplementary information, and signal and noise components are illustrated in Figs. S1 and S2.

### Univariate analysis

The preprocessed GM volume maps from the VBM analysis and the identified RSNs were used to investigate group differences between responders and non-responders. Age and total intracranial volume were entered as covariates for the VBM data, while only age was used as covariate for the RSN data. The significance level was set to *P* < 0.05 family-wise error (FWE) corrected and estimated using the threshold-free-cluster-enhancement statistic (TFCE)^45^ with permutation testing (10,000 permutations) using the TFCE toolbox (r167, http://dbm.neuro.uni-jena.de/tfce/) for the VBM data. For the resting-state data, the PALM toolbox (a112, https://fsl.fmrib.ox.ac.uk/fsl/fslwiki/PALM) was used since it allowed for permutation-based FWE correction across the whole-brain and all 48 RSNs at the same time. Both analyses accounted for two-tailed tests.

### Multivariate analysis

For the multivariate single-subject classification of responders and non-responders, we used the GM volume-maps from the VBM analysis and each RSN separately. The classification was performed using a Gaussian process classifier (GPC)^46^. Briefly, GPCs are multivariate Bayesian classifiers which allow to obtain valid probabilistic predictions by estimating the posterior distribution, given a pre-defined prior distribution. GPCs are a standard classifier used in machine learning for neuroimaging and has been shown to perform comparable to support vector machines^47^. We utilized the ability of the GPC’s to provide valid probabilistic predictions to investigate posthoc the performance of the classifier when a ‘reject’ option is implemented (see below). Univariate feature selection was performed on the training set to reduce the initial data dimension using nested 5-fold cross-validation (see Supplementary information). The performance was estimated by calculating sensitivity, specificity, balanced accuracy, area under the receiver-operator curve (AUC) and positive/negative predictive value (PPV/NPV) using ten times repeated 10-fold cross-validation to avoid overfitting bias. To estimate whether our classifier performed better than chance, label permutation tests with 1000 iterations were performed. The final *P*-values were Bonferroni corrected for 49 tests.

We also investigated the performance of the GPC when an uncertainty option was allowed: utilizing the probabilistic output of the classifier, we established regions of uncertainty for which the classifier would not make a prediction. For example, with an uncertainty region of 10% any probabilistic GPC output for a new patient which lies within 45–55%, would not be assigned a classification label (because the classification into responders and non-responders would be uncertain). Only patients with a higher (or lower) probability would be assigned to a class and considered for calculation of balanced accuracy. This allowed us to investigate how well our GPC would perform if classification has only to be made if a specific level of certainty is reached and how many patients would need to be excluded to reach that level.

The code used in the analysis of the data can be made available upon request.

## Results

### Clinical data

Demographic information, clinical variables and outcomes of statistical tests can be found in Table 1. There was no difference in demographics between the CC, responders or non-responders, nor any clinical difference between responders and non-responders at baseline. At follow-up non-responders showed a higher total CAPS score (t(42) = 7.89, *P* < 0.001) and higher use of serotonin reuptake inhibitors (*χ*^2^(1) = 5.77, *P* = 0.02).

**Table 1:** Demographics and clinical data.

	Combat Controls (*n* = 28)	Responders (*n* = 24)	Non-Responders (*n* = 20)	Test-value(df), *P*-value
Age (mean, SD [years])	37.00 (10.13)	33.25 (7.76)	38.65 (9.34)	F(2, 69) = 2.057, *P* = 0.136^a^
Gender (m/f)	28/0	24/0	20/0	
Handedness (left/ambidexter/right)	2/3/23	2/2/20	2/2/16	*χ*^2^(4) = 0.207, *P* = 0.995^b^
Education (median, IQR [ISCED])				
Own	6 [4.75, 7]	6 [5.75, 6]	5.5 [3, 6]	*χ*^2^(2) = 4.005, *P* = 0.135^c^
Mother	3.5 [2, 6]	3 [2, 4]	3 [2, 6]	*χ*^2^(2) = 1.325, *P* = 0.516^c^
Father	5 [2, 6.5]	3.5 [2.25, 7]	5 [2, 7]	*χ*^2^(2) = 0.044, *P* = 0.978^c^
Time since last deployment (mean, SD [years])	5.89 (6.56)	6.71 (7.83)	8.05 (9.51)	χ^2^(2) = 0.218, *P* = 0.897^c^
Number of times deployed (1/2/3/ > 3)	(10/8/4/6)	(9/5/3/7)	(8/3/6/2)	*χ*^2^(2) = 0.416, *P* = 0.812
FD (mean, SD)	0.10 (0.04)	0.09 (0.05)	0.12 (0.07)	*χ*^2^(2) = 3.278, *P* = 0.194^c^
TIV (mean, SD)		1550.02 (121.15)	1528.06 (166.44)	t(42) = −0.506, *P* = 0.616^d^
*Clinical scores at baseline*				
CAPS (mean, SD)		71.92 (15.06)	69.85 (11.45)	t(42) = 0.504, *P* = 0.617^d^
*Pre-treatment comorbid disorder baseline (SCID)*				
Mood disorder		13	10	*χ*^2^(1) = 0.076, *P* *=* 0.783^b^
Anxiety disorder		5	9	*χ*^2^(1) = 2.937, *P* *=* 0.087^b^
Somatoform disorder		1	1	*χ*^2^(1) = 0.017, *P* *=* 0.895^b^
*Pre-treatment medication*				
SRI		5	7	*χ*^2^(1) = 1.104, *P* *=* 0.293^b^
Benzodiazepines		7	3	*χ*^2^(1) = 1.247, *P* *=* 0.264^b^
Antipsychotics		2	0	*χ*^2^(1) = 1.746, *P* = 0.186^b^
Total number of treatment sessions (mean, SD)		9.86 (6.29)	10.05 (4.22)	t(38) = −0.114, *P* *=* 0.910^d^
*Received therapy*				
TF-CBT (yes/no)		6/18	10/10	*χ*^2^(1) = 2.946, *P* *=* 0.086^b^
EMDR (yes/no)		20/4	16/4	*χ*^2^(1) = 0.081, *P* *=* 0.775^b^
*Clinical scores at post-treatment*				
CAPS (mean, SD)		29.75 (16.53)	68.55 (15.89)	t(42) = 7.889, *P* *<* 0.001^d^*
*Post-treatment comorbid disorder post-treatment (SCID)*				
Mood disorder		3	3	*χ*^2^(1) = 0.096, *P* *=* 0.757^b^
Anxiety disorder		2	5	*χ*^2^(1) = 2.516, *P* = 0.113^b^
Somatoform disorder		0	1	*χ*^2^(1) = 1.293, *P* *=* 0.256^b^
Alcohol dependency		0	2	*χ*^2^(1) = 2.650, *P* = 0.104^b^
*Post-treatment medication*				
SRI		5	11	*χ*^2^(1) = 5.768, *P* = 0.016^b^*
Benzodiazepines		5	1	*χ*^2^(1) = 2.307, *P* = 0.129^b^
Antipsychotics		2	2	*χ*^2^(1) = 0.040, *P* = 0.841^b^

*SD* standard deviation*, IQR* interquartile range*, ISCED* international scale for education, *FD* framewise displacement, *TIV* total intracranial volume, *CAPS* clinician administered PTSD scale, *SCID* structured clinical interview for DSM IV Axis II disorders, *SRI* serotonin reuptake inhibitor, *TF-CBT* trauma-focused cognitive behavioral therapy, *EMDR* eye movement desensitization and reprocessing

^a^ANOVA

^b^*χ*^2^

^c^Kruskal–Wallis

^d^Two-sample *t*-test

*P < 0.05

### Univariate analysis

**Fig. 1:**
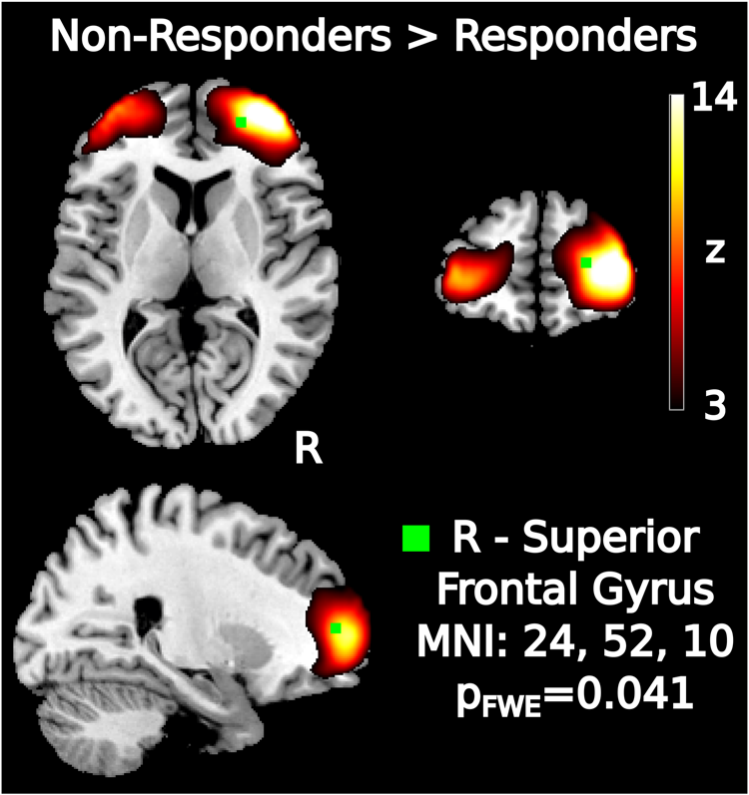
Results of the group-level univariate RSN analysis. Higher resting-state connectivity was observed in non-responders than responders in the frontopolar network. Two-tailed *P*-value was corrected for whole-brain comparisons and 48 networks.

After correction for multiple comparisons across all RSNs, the rs-fMRI analysis showed one network with significantly increased connectivity in non-responders as compared to responders (Fig. 1). The network was centered on the bilateral lateral frontal polar area and the difference was observed in the right superior frontal gyrus (*P*_FWE_ = 0.04). In Fig. S4, we show all univariate group-differences when no FWE-correction across networks was applied, performed for illustrative purposes only. No significant group differences in GM were observed.

### Multivariate analysis

**Fig. 2:**
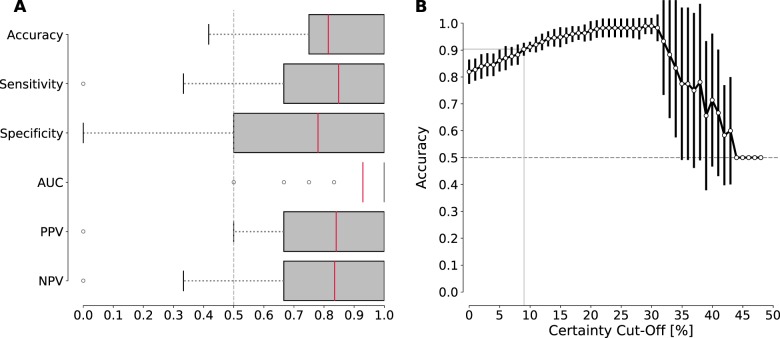
Results of the single-subject multivariate prediction analysis of treatment outcome. **a** The classification metrics of the pre-SMA network shown as box-and-whisker plots. Outliers plotted as circles were determined as values which lay outside 1.5 times the interquartile range. Please note that the box for the AUC metric collapsed because the first quartile and the median were the same value. **b** Posthoc evaluation of accuracy of the GPC classifier for various cut-off levels of probabilistic certainty. Calculations were performed for and averaged across the ten repetitions of the 10-fold cross-validation with SD plotted as error bars. For example, once 12 patients (27%) with low prediction certainty of 0.41–0.59 —where 0.5 is equal probability of prediction— would be excluded, accuracy would increase to over 90%.

GPC’s trained on a network centered around the pre-supplementary motor area (pre-SMA) could classify non-responders and responders with an average cross-validated balanced accuracy of 81.4% (SD: 17.2, *P*_Bonferroni_ < 0.05) (Fig. 2a). The network showed excellent AUC (0.929, SD: 0.149) with high sensitivity (84.8%, SD: 25.1), moderately high specificity (78% SD: 28.6), and high PPV/NPV (0.840/0.835, SD: 0.214/0.262). No other network showed significant classification performance after Bonferroni correction was applied, including the network that showed a significant difference on the group-level in the univariate analysis. However, if no Bonferroni correction is applied this network becomes significant, as well as three additional networks. Uncorrected networks and consistently selected features are shown for illustrative purposes in Fig. S5.

**Fig. 3:**
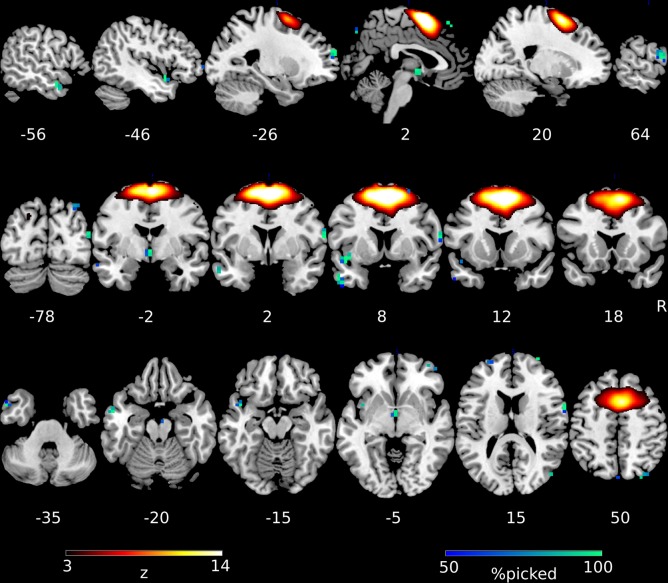
Best performing network in the multivariate classification (pre-SMA) in hot colors and the most often selected voxels during the classification in cold colors.

To investigate which regions of the pre-SMA network were most important for the classification process we examined consistently selected voxels during the feature selection process. We tracked the selection frequency of voxels across cross-validation runs, looking at voxels which were selected in >50% of the runs (Table 2 and Fig. 3). Regions in both hemispheres located outside the group-network were contributing to the classification performance. The largest clusters were located in the left inferior temporal gyrus (n_voxel_ = 14), left superior frontal gyrus (n_voxel_ = 10), and right precentral gyrus (n_voxel_ = 9). For illustrative purposes we also computed mean correlations for responder and non-responder groups separately between average time-courses of the largest clusters(n_voxel_ > 5, Table 2) and the subject-specific time-courses of the pre-SMA network identified by dual regression (Fig. S3). Patterns of positive, negative and no significant connectivity with the network can be observed. Note that null-connectivity voxels might still contribute to the classification by removing common noise sources from the overall pattern^48^.

**Table 2:** Most frequently selected features during the nested-cross-validation procedure of the pre-SMA network.

Number of voxels	Max frequency within cluster (%)	MNI coordinates of max value (mm)	Region name
14	99	−52, 8, −34	Left inferior temporal gyrus
10	100	−24, 60, 22	Left superior frontal gyrus
9	100	64, 4, 14	Right precentral gyrus
7	100	−44, 8, −14	Left insula, left superior temporal pole
6	93	28, −80, 50	Right superior parietal lobule
6	100	0, −4, −2	Hypothalamus
4	98	0, 36, 58	Left medial frontal gyrus
4	89	32, 64, 6	Right middle frontal gyrus
4	96	48, −76, 18	Right middle occipital gyrus
2	92	0, −80, 46	Left precuneus
2	76	40, −84, 26	Right middle occipital gyrus
2	67	−44, 56, 2	Left middle frontal gyrus
2	75	48, 52, −6	Right middle orbitofrontal gyrus
2	63	36, 44, −18	Right inferior orbitofrontal gyrus
1	84	40, 56, −6	Right middle orbitofrontal gyrus
1	100	32, 64, 14	Right superior frontal gyrus
1	67	−4, 68, −10	Left medial orbitofrontal gyrus
1	57	4, −88, 34	Left cuneus
1	69	28, 8, 66	Right superior frontal gyrus

Additionally, we provided a posthoc evaluation of what would happen if prediction would only be made for patients for which a high degree of certainty of the classifier is established. As illustrated in Fig. 2b, this ability to ‘reject’ patients from the classification with increasing classification certainty leads to increasing accuracy while at the same time reducing the number of patients for which the GPC can make a classification. For example, once 12 patients (27%) with low prediction certainty of 0.41–0.59—where 0.5 is equal probability of prediction—would be excluded, accuracy would increase to over 90%.

## Discussion

The present study investigated the possibility of using pre-treatment structural MRI and rs-fMRI data to predict the response to trauma-focused psychotherapy in male combat veterans with PTSD. The results showed that rs-fMRI data successfully distinguished between responders and non-responders in univariate and multivariate analyses. The univariate analysis detected group differences in a network centered on the frontal pole, and the multivariate analysis predicted treatment response on an individual level using pre-SMA connectivity with an accuracy of 81.4%. Whereas previous studies have focused on MRI-based treatment outcome predictors at the group level, our results suggest that single-subject prediction is also feasible. This result provides a proof-of-concept for the feasibility of developing predictive biomarkers, which could enable personalized treatment for patients with PTSD.

Our multivariate analysis revealed the predictive importance of the pre-SMA. This brain area is closely linked to the SMA, and is involved in motor preparation, response inhibition, and imagination^49–51^. Intriguingly, resting-state connectivity within this network is also predictive for the response to electroconvulsive therapy in depression^52^. The main difference in results is that the network in the current study is more confined to the pre-SMA due to the use of ICA with 70 components instead of 32 components, which was associated with a larger network that consisted of a large part of the dorsomedial prefrontal cortex. Together, this suggests that pre-SMA connectivity may determine responsiveness to treatment, regardless of intervention and disorder.

The discovered network is different from the ventral attention network (VAN, consisting of the insula, dorsal anterior cingulate, anterior middle frontal gyrus, and supramarginal gyrus) that was recently reported. The VAN in combination with delayed recall performance in a verbal memory task could predict prolonged exposure therapy outcome in a sample of ~19 civilians with PTSD with sensitivity and specificity ≥80%^25^. But even though both studies used rs-fMRI, the underlying biomarkers cannot readily be compared. First, the variables tested in^25^ were discovered by performing comparisons between healthy controls and PTSD patients, whereas we discovered the pre-SMA network from comparisons between responders and non-responders directly. Second, the authors did not investigate any other networks beyond the VAN for treatment outcome prediction. And third, the brain regions that are part of the VAN were part of distinct RSNs in our ICA analysis, whereas the VAN was considered one network in the previous study. Therefore, it remains to be tested whether VAN or pre-SMA connectivity is also predictive in other samples. Regardless, both studies demonstrate that rs-fMRI contains information that is informative for predicting psychotherapy outcome on an individual level.

The univariate group analysis showed increased connectivity in non-responders in the frontal pole. The frontal pole region (BA 10) has been implicated in a multitude of cognitive tasks, including attention, perception, language, and memory tasks^53,54^. Specifically, the lateral parts of the frontal pole are more associated with working memory and episodic memory retrieval while medial parts of the frontal pole were mostly involved in mentalizing, which is the reflection of your own emotions and mental states^53,54^. This division of the frontal pole was recently confirmed by a cytoarchitectonic parcellation indicating two distinct areas: a more lateral frontal pole area 1 (FP 1) and a more medial frontal pole area 2 (FP 2). Our frontal polar network was mostly located in FP 1 and may, therefore, be primarily associated with memory-related processes.

The difference between the identified networks in the univariate and multivariate analyses might seem counterintuitive at first but can be explained by the differences in objective and methodology of both analyses. This discrepancy is in line with the observation that significant group-level differences do not necessarily translate to high classification accuracies because of strongly overlapping distributions and different goals of the analysis^18,19^. A significant *P*-value in a group-level analysis does not have to correspond to the ability of distinguishing between individual patients because the statistically significant difference in average values might show low effect sizes. In these case classification performance will be low. In addition, the goal of statistical inference is the identification of localized differences between groups while the goal of classification is to find the best multivariate combination of data, which would allow to generalize the effect to new subjects. These are two inherently different goals which therefore can lead to different outcomes.

In contrast to our results, previous studies that have used univariate analysis of structural MRI and task-based fMRI data have primarily pointed to pre-treatment differences in the anterior cingulate cortex, amygdala, hippocampus, and insula^13–17,32–35^. However, direct comparison with our study is difficult since there are numerous differences between our study and those previous studies. For example, most studies that investigated structural MRI data used a predefined region of interest approach^26,32,33,35^ instead of a whole-brain approach. Most fMRI studies have investigated task-induced changes instead of investigating resting-state fMRI recordings^13–17^. And finally, different types of psychotherapies (such as prolonged exposure therapy), different PTSD populations and experienced trauma, and different treatment-criteria make a direct comparison challenging^55^.

This can be exemplified with the absence of results for the structural MRI analysis which is in contrast to our previous finding of differences in hippocampal volume between patients with remitted vs. persistent PTSD^26^. This difference could be due to the calculation of the volumes: in the present study, a VBM analysis was employed to provide a highly multivariate data set that could be optimally used during the classification procedure, whereas we previously estimated hippocampal volume using segmentation in Freesurfer. In addition, in this study we chose to focus on treatment response while previously we investigated the more stringent criterion of treatment remission to focus on PTSD persistence. Finally, the previous study was employing a repeated-measures design combining pre- and post-treatment data while the current study only focused on the pre-treatment data.

The current study has several limitations. The sample size in the current study is small for a machine learning application. This could result in high variance of the estimated accuracy and the results, therefore, require further validation and replication in independent samples^56^. Another limitation of this study is the use of an all-male veteran sample. This limits the generalization of the results to other patients with PTSD. Therefore, a replication of the proposed approach in a more diverse sample would be desirable. Finally, the treatments received by the patients represent a heterogeneous mix of different trauma-focused psychotherapies. While they are considered as first-line treatments and the fact that in realistic settings multiple treatments might be employed by therapists, the results are not specific to one particular treatment. Therefore, the current approach might obscure specific individual patient-by-treatment interactions. Future studies should aim to determine the most optimal treatment for each patient.

In conclusion, the current study shows that treatment response to trauma-focused psychotherapy can be predicted for individual patients with PTSD using machine learning analysis of rs-fMRI data. This proof-of-concept study demonstrates the feasibility to develop neuroimaging biomarkers for treatment response, which will enhance the personalized treatment of patients with PTSD.

## Supplementary information

Supplementary Methods
