# Supplementary material for: Individual prediction of psychotherapy outcome in posttraumatic stress disorder using neuroimaging data
Source: Transl Psychiatry. 2019 Dec 2;9:326. doi: 10.1038/s41398-019-0663-7 (PMC6889413; doi:10.1038/s41398-019-0663-7)
Supplement: Supplementary file 1 — Supplementary Methods [file 41398_2019_663_MOESM1_ESM.docx]

**Supplementary Material**

**Individual Prediction of Psychotherapy Outcome in Posttraumatic Stress Disorder using Neuroimaging Data**

Paul Zhutovsky (MSc)^1,2^, Rajat M. Thomas (PhD)^1,2^, Miranda Olff (PhD)^1,3^, Sanne J.H. van Rooij (PhD)^4^, Mitzy Kennis (PhD)^5^, Guido A. van Wingen (PhD)^1,2*^, Elbert Geuze (PhD)^6,7*^
^1^Amsterdam UMC, University of Amsterdam, Department of Psychiatry, Amsterdam Neuroscience, Amsterdam, The Netherlands

^2^Amsterdam Brain and Cognition, University of Amsterdam, Amsterdam, The Netherlands
^3^Arq Psychotrauma Expert Group, Diemen, The Netherlands

^4^Department of Psychiatry and Behavioral Sciences, Emory University School of Medicine, Atlanta, GA, USA

^5^Clinical Psychology Department, Utrecht University, Utrecht, The Netherlands

^6^Utrecht University Medical Center, Rudolf Magnus Institute of Neuroscience, Utrecht, The Netherlands

^7^Brain Research and Innovation Center, Ministry of Defense, Utrecht, The Netherlands

*Equally contributing authors

**Supplementary Materials and Methods**

**MRI processing**

The voxel-based morphometry (VBM) analysis was performed using the SPM12 toolbox (v7219). Briefly, we obtained gray matter (GM) segmentations applying the unified segmentation approach^1^. The GM maps were then normalized to MNI space (1.5mm^3^) based on DARTEL registration^2^ using a template derived from 555 healthy controls of the IXI-database ([http://www.brain-development.org](http://www.brain-development.org/)) in MNI space provided by the CAT12 toolbox (<http://www.neuro.uni-jena.de/cat/>). The normalized GM images were modulated by the Jacobian determinant to preserve local tissue volume and spatially smoothed with a kernel of 8mm at FWHM.

**fMRI processing**

Preprocessing of fMRI images was performed using the advanced normalization tools (ANTs, 2.1.0, <http://stnava.github.io/ANTs/>)^3^ and FMRIB Software Library (FSL, 5.0.10)^4^. For the purpose of registration to MNI space and extraction of white matter (WM) and cerebral spinal fluid (CSF) signal from fMRI scans, T1 images were bias-field corrected using the N4 algorithm^5^ and brain-extracted using scripts from the ANTs toolbox and the Oasis template (<https://www.oasis-brains.org/>). Images were then segmented into GM, WM and CSF partial volume estimates using FSL’s FAST^6^. The skull-stripped images were normalized to MNI space using the ANTs symmetric normalization procedure^3^. One PTSD patient was excluded based of an artifact in his MRI scan.

fMRI image preprocessing consisted of realignment, co-registration to the T1 image using boundary-based registration^7^ and spatial smoothing with a 8mm FWHM kernel. Motion has a strong effect on resting-state fMRI measures^8^ and therefore has to be addressed further during the preprocessing of rs-fMRI data. Therefore, we calculated framewise displacement (FD)^9^ of the raw data and excluded subjects based on the following criteria: (1) any rotation/translation parameter >4mm/◦, (2) average FD > 0.45, (3) more than 150 volumes with an individual FD of 0.25, leading to less than 4min of motion free rs-fMRI data^10^. Applying these criteria led to the exclusion of three PTSD patients and one combat control. The remaining patients did not differ in their motion levels (see Table 1). Furthermore, motion was additionally addressed by applying ICA-AROMA^11^ to automatically identify single-subject ICA components associated with motion. These components were then regressed out from the data. Further structured noise was removed by performing nuisance regression with average WM and CSF signals. For that the calculated WM/CSF segmentations of the T1 image were transformed to EPI space and thresholded conservatively at 0.95. The denoised fMRI images were transformed to MNI space at 4mm and high-pass filtered at 0.01Hz.

**Meta-ICA**

For the meta-ICA we repeatedly (25 times) extracted 20 participants out of the 28 combat controls at random and performed a temporally-concatenated group-ICA with the number of components fixed to 70. The obtained spatial maps (25 * 70 = 1750) were merged and entered into an additional (meta-)ICA with 70 components. The number of components was determined because it was shown to provide good insight into clinical differences of patient groups^12^. Following the meta-ICA, group spatial components were investigated visually and using an automatic approach verifying their reproducibility across individual ICA runs and the proportion of the components located in the gray matter^13^. 48 components were identified as carrying non-noise related resting-state activity (Supplementary Figure 1 and Supplementary Figure 2). Following the identification of the components dual regression was performed to identify subject specific spatial maps corresponding to the group components^14^. Dual regression was applied using the group maps computed through meta-ICA on the combat controls and the rs-fMRI data of the PTSD patients.

**Multivariate Analysis**

Classification was performed using a Gaussian process classifier (GPC). We chose a zero mean and a normalized linear kernel function for the prior distribution following recommendations in the field^15^. To infer the parameters of the posterior distribution we used a Probit likelihood function with the expectation maximization algorithm for inference^16^. To reduce the initial dimensionality of the classification problem univariate feature selection was performed. For that we computed the average univariate difference between connectivity values in every voxel using only participants of the training set. The difference was then z-scaled and thresholded. To determine the optimal threshold we investigated z-values from 2.5 until 4.0 in steps of 0.1 using nested 5-fold cross-validation on the training set. The optimal value was chosen as the one which generated the highest average balanced accuracy (average between sensitivity and specificity) across the five folds. The GPC was implemented using the Python (version 2.7.15) interface of the Shogun machine learning toolbox (version 6.1.3, <http://shogun-toolbox.org/>).


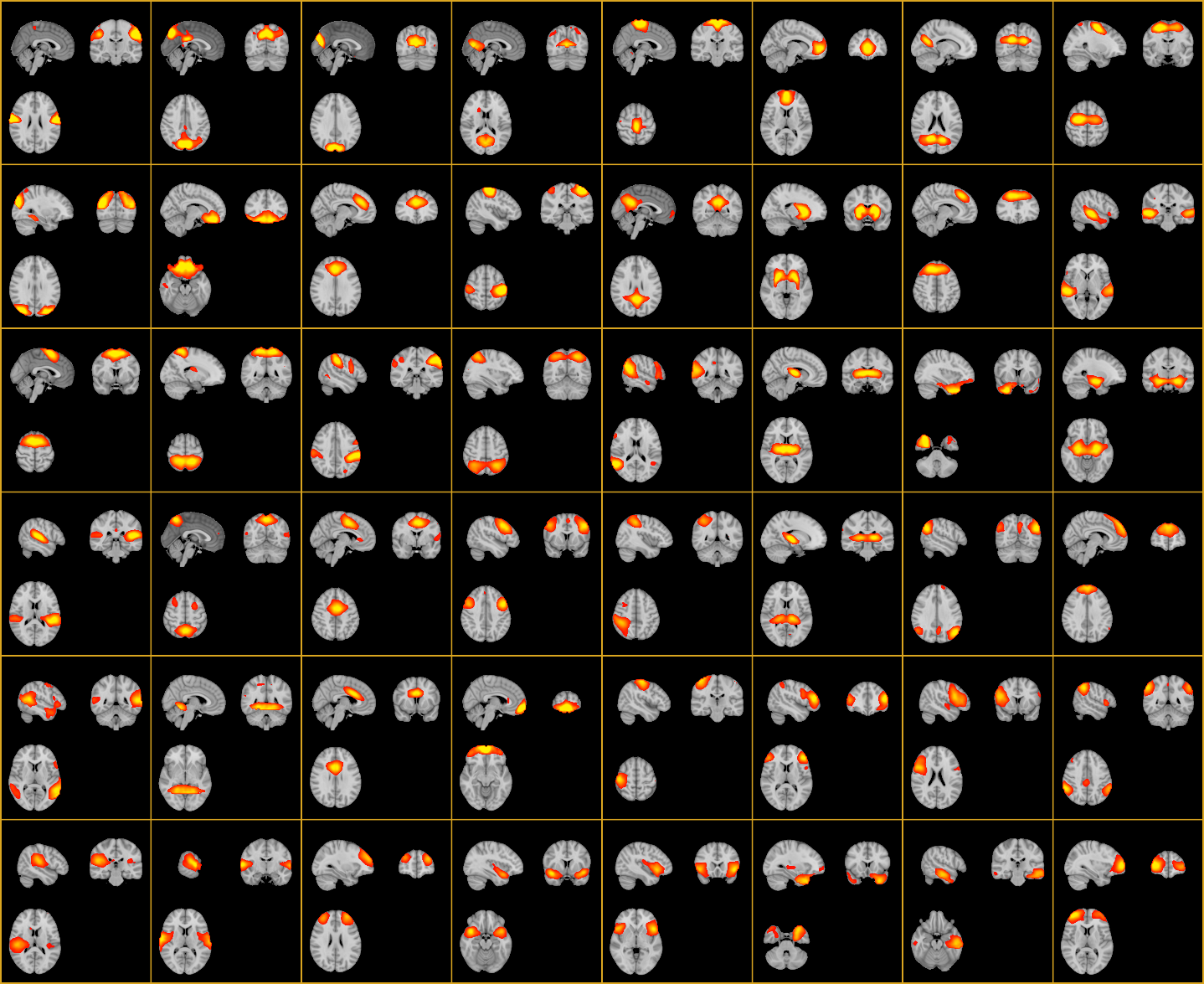


*Figure S1.* 48 out of 70 networks calculated via a meta-ICA approach which were identified as carrying signal-related information


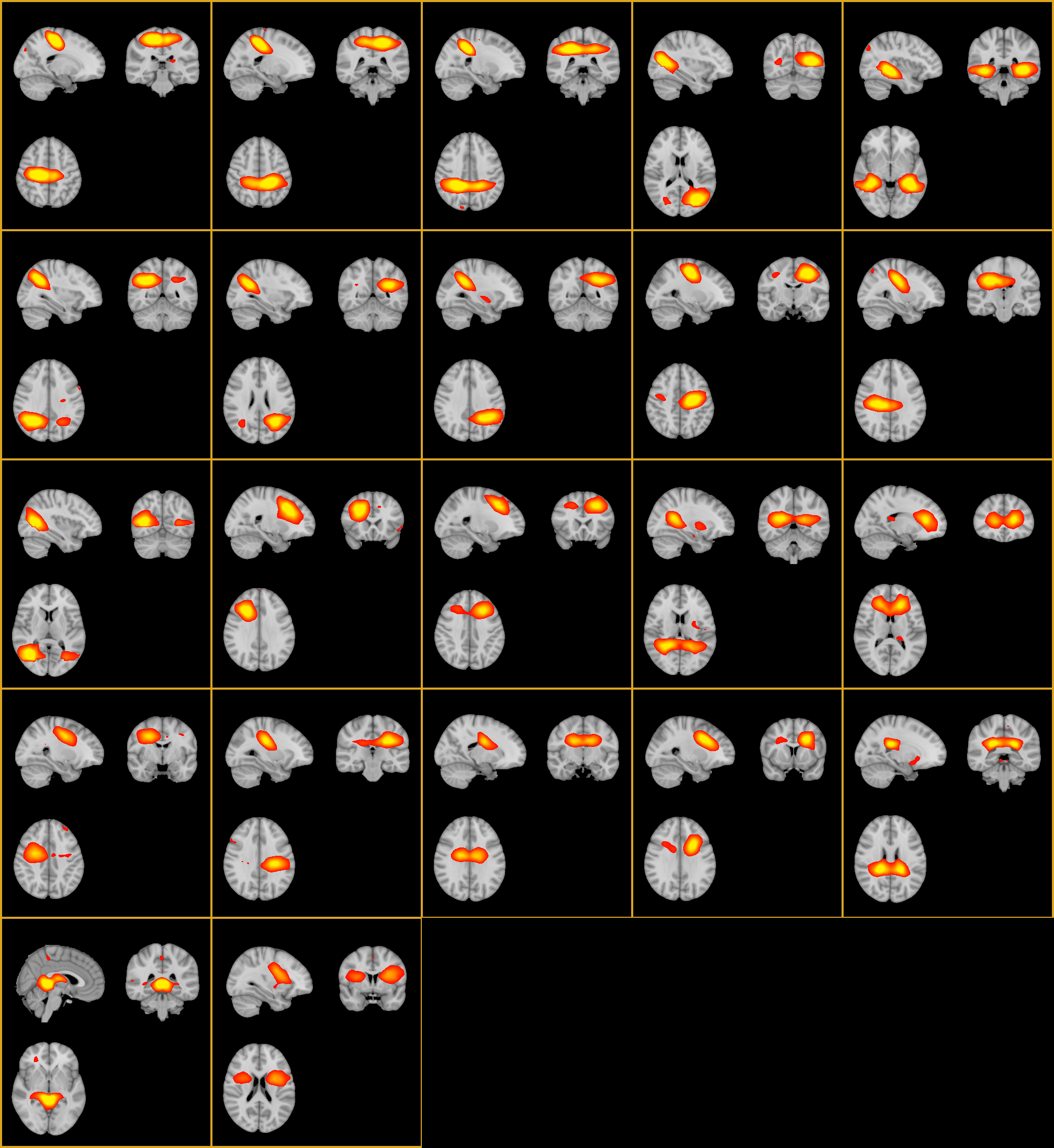


*Figure S2.* 22 out of 70 meta-ICA networks which were related to noise sources.

**
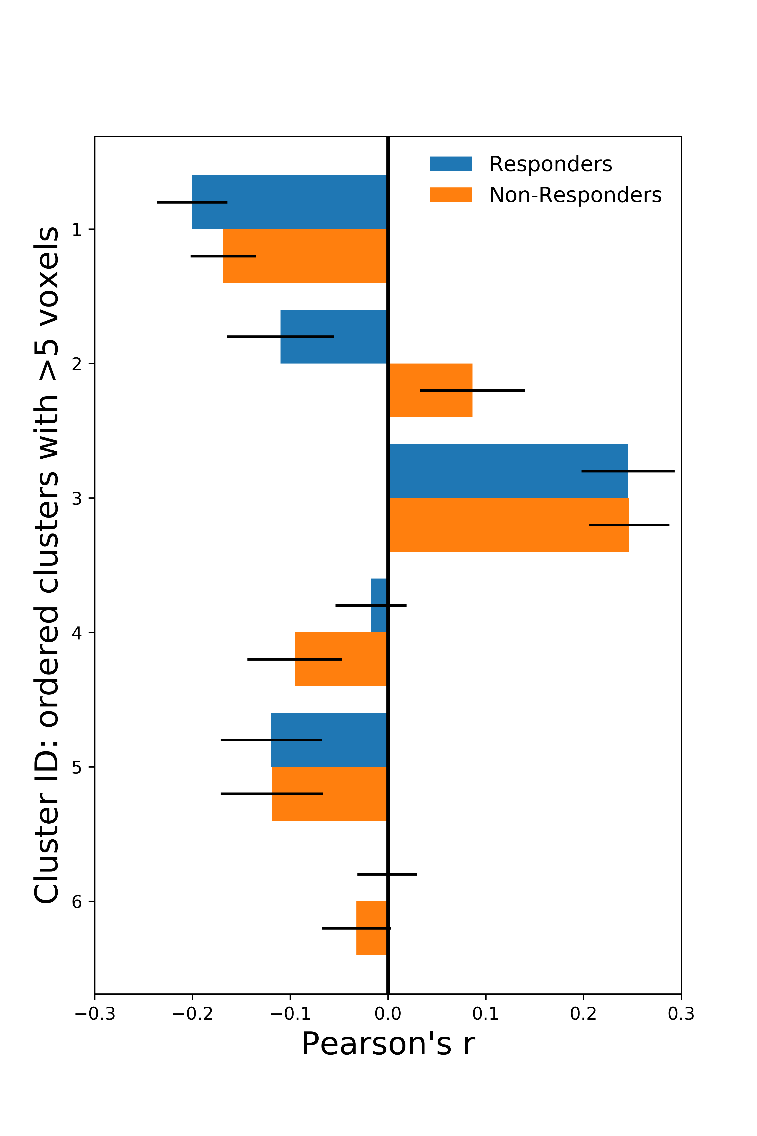
**

*Figure S3.* Mean correlation across patient groups between average time-courses extracted from the largest clusters (>5 voxels) of consistently selected features (see Table 2) with the pre-SMA time-courses of each participant (extracted via dual regression). Errorbars show standard error of the mean.

**
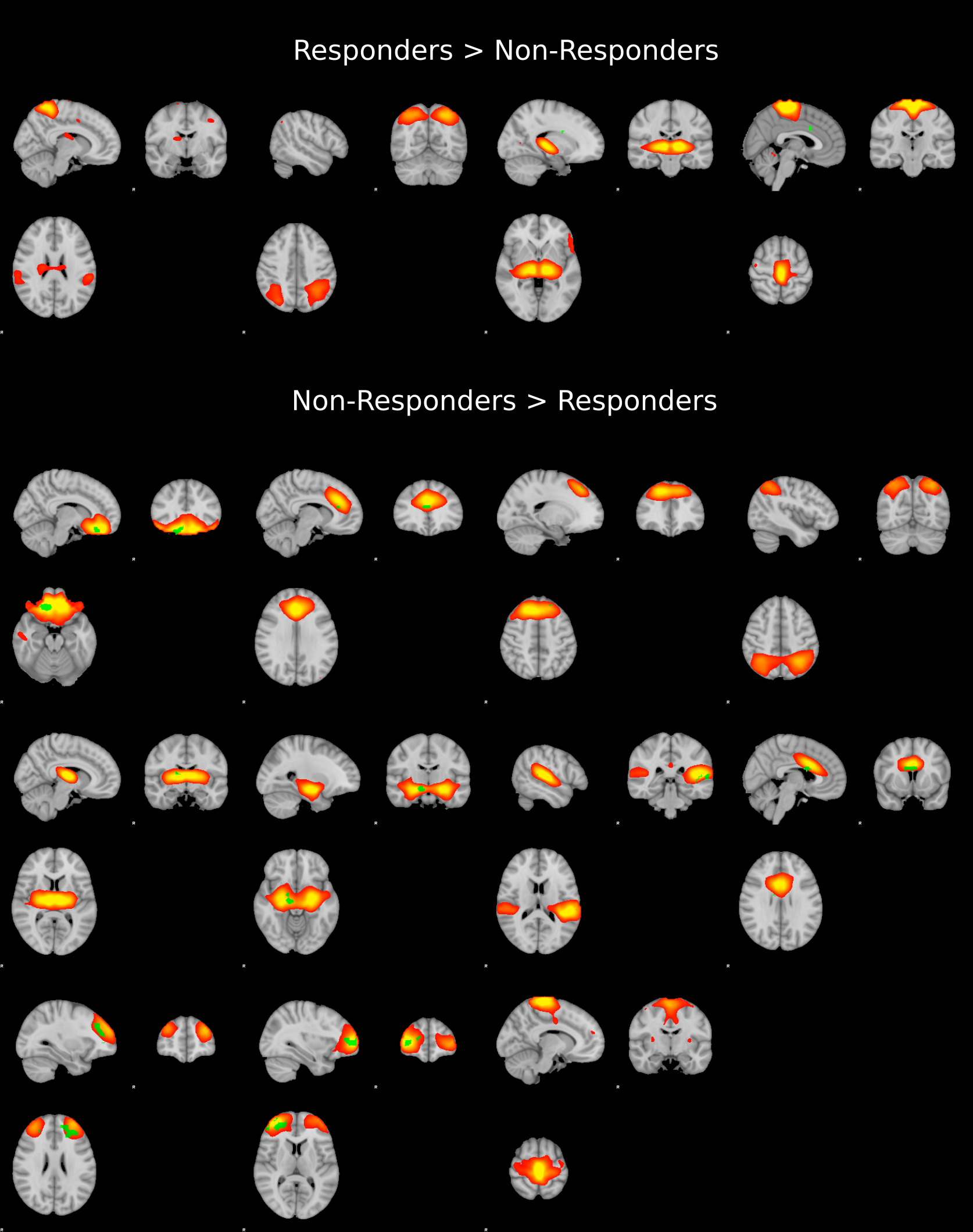
**

*Figure S4*. Results of the univariate group analyses showing differences between responders and non-responders when *no* further correction for the investigated networks is performed. This visualization is purely exploratory and inflated Type-1 error rates have to be assumed. Right side of the brain is plotted on the left. The RSNs (hot colors) were thresholded at z 3-14. The significant group-differences are plotted in green.


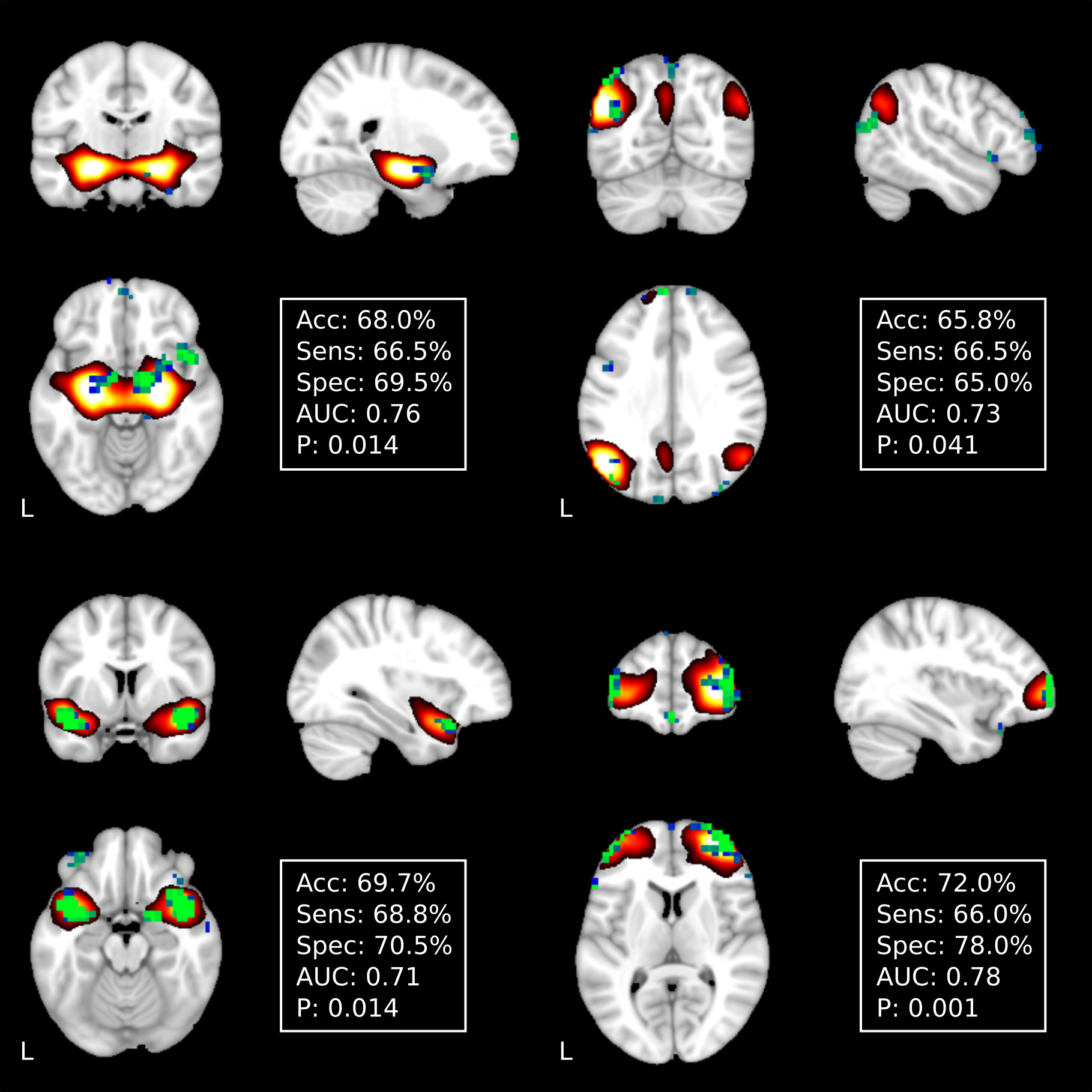


*Figure S5.* Results of the multivariate analysis if the final P-value is not adjusted according to Bonferroni correction for the number of RSNs tested. These results are therefore purely exploratory and might show inflated Type-I error. RSNs are plotted in hot colors and were thresholded at z 3-14. The consistently selected features (>50% selected across cross-validation iterations) during the classification are plotted in blue to green colors.

**References**

1. Ashburner J, Friston KJ. Unified segmentation. *Neuroimage* 2005; **26**(3)**:** 839-851.

2. Ashburner J. A fast diffeomorphic image registration algorithm. *Neuroimage* 2007; **38**(1)**:** 95-113.

3. Avants BB, Epstein CL, Grossman M, Gee JC. Symmetric diffeomorphic image registration with cross-correlation: evaluating automated labeling of elderly and neurodegenerative brain. *Med Image Anal* 2008; **12**(1)**:** 26-41.

4. Jenkinson M, Beckmann CF, Behrens TE, Woolrich MW, Smith SM. Fsl. *Neuroimage* 2012; **62**(2)**:** 782-790.

5. Tustison NJ, Avants BB, Cook PA, Zheng Y, Egan A, Yushkevich PA*, et al*. N4ITK: improved N3 bias correction. *IEEE Trans Med Imaging* 2010; **29**(6)**:** 1310-1320.

6. Zhang Y, Brady M, Smith S. Segmentation of brain MR images through a hidden Markov random field model and the expectation-maximization algorithm. *IEEE Trans Med Imaging* 2001; **20**(1)**:** 45-57.

7. Greve DN, Fischl B. Accurate and robust brain image alignment using boundary-based registration. *Neuroimage* 2009; **48**(1)**:** 63-72.

8. Ciric R, Wolf DH, Power JD, Roalf DR, Baum GL, Ruparel K*, et al*. Benchmarking of participant-level confound regression strategies for the control of motion artifact in studies of functional connectivity. *Neuroimage* 2017; **154:** 174-187.

9. Jenkinson M, Bannister P, Brady M, Smith S. Improved optimization for the robust and accurate linear registration and motion correction of brain images. *Neuroimage* 2002; **17**(2)**:** 825-841.

10. Van Dijk KR, Hedden T, Venkataraman A, Evans KC, Lazar SW, Buckner RL. Intrinsic functional connectivity as a tool for human connectomics: theory, properties, and optimization. *J Neurophysiol* 2010; **103**(1)**:** 297-321.

11. Pruim RHR, Mennes M, van Rooij D, Llera A, Buitelaar JK, Beckmann CF. ICA-AROMA: A robust ICA-based strategy for removing motion artifacts from fMRI data. *Neuroimage* 2015; **112:** 267-277.

12. Abou Elseoud A, Littow H, Remes J, Starck T, Nikkinen J, Nissila J*, et al*. Group-ICA Model Order Highlights Patterns of Functional Brain Connectivity. *Front Syst Neurosci* 2011; **5**(37)**:** 37.

13. Cerliani L, Mennes M, Thomas RM, Di Martino A, Thioux M, Keysers C. Increased Functional Connectivity Between Subcortical and Cortical Resting-State Networks in Autism Spectrum Disorder. *JAMA Psychiatry* 2015; **72**(8)**:** 767-777.

14. Beckmann CF, Mackay CE, Filippini N, Smith SM. Group comparison of resting-state FMRI data using multi-subject ICA and dual regression. *Neuroimage* 2009; **47**(Suppl 1)**:** S148.

15. Marquand A, Howard M, Brammer M, Chu C, Coen S, Mourao-Miranda J. Quantitative prediction of subjective pain intensity from whole-brain fMRI data using Gaussian processes. *Neuroimage* 2010; **49**(3)**:** 2178-2189.

16. Minka TP. A family of algorithms for approximate Bayesian inference, Massachusetts Institute of Technology, 2001.
